# Supplementary material for: Value of 3D printing technology combined with indocyanine green fluorescent navigation in complex laparoscopic hepatectomy
Source: PLoS One. 2022 Aug 11;17(8):e0272815. doi: 10.1371/journal.pone.0272815 (PMC9371281; doi:10.1371/journal.pone.0272815)
Supplement: Attachment — Submitted filename: Response to Reviewers.docx [file pone.0272815.s001.docx]

Dear Prof. Gianfranco D. Alpini,

Thank you very much for your letter and advice. We have revised the manuscript, and would like to re-submit it for your consideration. We have addressed the comments raised by the reviewers, and the amendments are highlighted in the revised manuscript. Point by point responses to the reviewers’ comments are listed below this letter. This manuscript has been edited and proofread in standard English. We hope that the revised version of the manuscript is now acceptable for publication in your journal.
I look forward to hearing from you soon.

With best wishes,

Yours sincerely,
Chengwu Zhang
Corresponding author

We would like to express our sincere thanks to the reviewers for the constructive and positive comments.
Replies to Reviewer #1
Specific Comments

1. Thorough editing of the manuscript language. Various grammar errors were found in this manuscript and should be reviewed. Notably, words should not start with conjunction words like " But".
   Answer: The manuscript has been edited in an intelligible fashion and written in standard English.
2. The discussion should be rewritten (first couple of paragraphs) due to its similarity to the introduction. The discussion is where the authors are able to criticize their work and see how it supports/contradicts other literature. It is this reviewers suggestion that the authors revamp this part of their work.
   Answer: The discussion has been rewritten in the first couple of paragraphs in the revised version to address this issue.
3. Inclusion of the cost effectiveness of 3D printing + Dye insertion should be included in this manuscript. What are the potential healthcare costs of these difficult surgeries? How much does inclusion of 3D visualization and dye injection help reduce this cost both for clinicians and patients?

Answer: Yes, the relevant data were added to state the cost effectiveness of 3D printing and ICG imaging in laparoscopic hepatectomy in the Table 2. in the revised version.

Replies to Reviewer #2
Specific Comments

1. Data on the presence of liver cirrhosis in liver parenchyma should be provided.
2. Occurrence of liver decompensation at 30-day and 90- day post-intervention should be provided.

Answer: The relevant data were added in the Table 1 and 2 in the revised manuscript.

3. Authors should carefully discuss how much £D printing(3DP) technology and indocyanine green(ICG) fluorescent navigation may imply in term of health costs.
   Answer: Discussion about cost effectiveness of 3DP and ICG fluorescent navigation has been added in the revised version.
4. Value of liver tests may vary among the groups in post surgical period. In particular cholestasis indexes. Please provide these data.

Answer: The relevant data were provided in the Table 2 in the revised manuscript.

5. English should be revised.

Answer: The manuscript has been edited in an intelligible fashion and written in standard English.
