# Supplementary material for: Value of 3D printing technology combined with indocyanine green fluorescent navigation in complex laparoscopic hepatectomy
Source: PLoS One. 2022 Aug 11;17(8):e0272815. doi: 10.1371/journal.pone.0272815 (PMC9371281; doi:10.1371/journal.pone.0272815)
Supplement: Attachment — Submitted filename: Response to Reviewers.docx [file pone.0272815.s002.docx]

Dear reviewers,

Thank you very much for your constructive and positive comments. Some of these suggestions are exactly what our article's issues are. We have revised the manuscript point by point as follows. We hope that the revised version of the manuscript is now acceptable for publication.

Replies to Reviewer
Specific Comments

**1.Further information about the tumor stages, unifocality or multifocality (HCC), and distance to the margins (R0) and why they had** **2 cases with positive margins? How were the complications managed? Was a drain sufficient for the bile leak?**

Answer: All the HCCs were single solid tumors, the further information was collected and added in the Table 2. in the revised version. 2 cases with positive margins(<2mm) were found in the Non-3DP group at the final histopathology of surgical specimen, that's because the two HCCs were adjacent tightly to important vessels that must be preserved. They were treated by salvage TACE and oral lenvatinib. The patients suffered from bile leak were only treated by keeping drain and had an uneventful recovery with no more complications eventually. Abdominal drainage tube was usually removed about one month after discharge.

**2.Total duration of Pringle maneuver(s) for both cohorts would be** **interesting to compare and evaluate its impact on** **LFTs.**

Answer: Yes, it was interesting. LFTs on the day 1, 3, 5 after operation actually showed no difference between the two groups. The relevant data listed in the Table 2.

**3.The introduction and discussion are thorough however several grammatical and spelling errors need to be** **corrected and the manuscript would benefit from editorial input from a native English speaking person. The authors mention the limitation of their study from an oncological outcomes standpoint. Do they have any results on at least a 6 months - 2 year follow up, given that the patients were included from 2019?**

Answer: The manuscript has been corrected in standard English. Follow-up data were collected and added in the Table 2. in the revised version. There was no difference in overall survival(OS) (*P*=0.75) and recurrence-free survival (RFS) (*P*=0.25) between the two groups in patients with HCC.

**4.In table 1 the authors mention p values >0.05 for preoperative diagnosis and lesion location. Why did the authors not include the full number as** **the p value is greater than 0.05, hence not significant?**

Answer: Thank you for your meticulous review, the *p* value was calculated by SPSS and corrected.

**5.In patients and methods, the authors state "Written informed consent was obtained from all study participants. Due to the retrospective nature of this study, patient consent for inclusion was waived." These two statements are** **contradictory and the authors need to clarify what was done, whether patients consented or if the consent process was waived.**

Answer: Thank you for your meticulous review again, the mistake should not happen. We deleted the contradictory sentence“Written informed consent was obtained from all study participants.”

With best wishes,

Yours sincerely,
Chengwu Zhang
Corresponding author
