# Supplementary material for: Value of 3D printing technology combined with indocyanine green fluorescent navigation in complex laparoscopic hepatectomy
Source: PLoS One. 2022 Aug 11;17(8):e0272815. doi: 10.1371/journal.pone.0272815 (PMC9371281; doi:10.1371/journal.pone.0272815)
Supplement: Attachment — Submitted filename: Response to Reviewers.docx [file pone.0272815.s003.docx]

Dear reviewers,

Thank you very much for your constructive and positive comments. We have revised the manuscript point by point as follows. This manuscript has been edited and proofread in standard English. We hope that the revised version of the manuscript is now acceptable for publication.

Replies to Reviewer 3

Specific Comments from Reviewer #3: The manuscript PONE-D-21-33418R2 has been significantly improved and the data presentation as well. There are still too many spelling / grammatical issues in the manuscript to accept it for publication. I recommend to have a native English speaking person assist the authors with editing the manuscript. The data is interesting however there are no significant differences between the two analyzed patient groups to make any significant recommendation.

Answer: The manuscript has been edited in an intelligible fashion and written in standard English. Yes, this modified procedure didn't improve the intraoperative and short-term outcomes, but the optimal surgical strategy for complex laparoscopic hepatectomy can be chosen with the help of 3DP technology and ICG fluorescent navigation.

Replies to Reviewer 4

Specific Comments from Reviewer #4: I did not have the opportunity to review the earlier version of this manuscript, but this one reads well and was very interesting. The authors are to be commended for their innovative approach to complex laparoscopic hepatectomy; I think their work will be of broad interest to hepatobiliary surgeons worldwide, despite a lack of statistically significant improvements in surgical outcome in their cohort of patients. The rationale behind my recommendation lies in that I think this manuscript would be of greater interest to a surgical journal, and would be seen by more surgeons in that type of publication.

Answer: Thank you for the approval of our work and encouragement. We have been very impressed by the overall quality of the work published by PLOS ONE. It will be my great pleasure and honor to publish our novel work in the PLOS ONE as well. Thank you very much for your kind recommendation.

With best wishes,

Yours sincerely,
Chengwu Zhang
Corresponding author
